# Assessing the Presence of *Pithomyces chartarum* in Pastureland Using IoT Sensors and Remote Sensing: The Case Study of Terceira Island (Azores, Portugal)

**DOI:** 10.3390/s24144485

**Published:** 2024-07-11

**Authors:** Mariana Ávila, João Pinelo, Enrique Casas, César Capinha, Rebecca Pabst, Iga Szczesniak, Elizabeth Domingues, Carlos Pinto, Valentina Santos, Artur Gil, Manuel Arbelo

**Affiliations:** 1 Atlantic International Research Centre, 9700 Angra do Heroísmo, Portugal; mariana.avila@aircentre.org (M.Á.);; 2 Departamento de Física, Universidad de La Laguna, 38200 San Cristóbal de La Laguna, Spain; 3 Centro de Estudos Geográficos, Instituto de Geografia e Ordenamento do Território da Universidade de Lisboa, Universidade de Lisboa, 1600 Lisbon, Portugal; 4 Laboratório Associado Terra, 1349 Lisbon, Portugal; 5 Global Health and Tropical Medicine, Instituto de Higiene e Medicina Tropical, Universidade Nova de Lisboa, 1349 Lisbon, Portugal; 6 UNICOL-Cooperativa Agrícola, C.R.L., 9700 Angra do Heroísmo, Portugal; 7 Faculdade de Ciências Agrárias e do Ambiente (FCAA), Universidade dos Açores, 9700 Angra do Heroísmo, Portugal; 8 Laboratório Regional de Veterinária, 9700 Angra do Heroísmo, Portugal; 9 Instituto de Investigação em Vulcanologia e Avaliação de Riscos (IVAR), Universidade dos Açores, 9500 Ponta Delgada, Portugal

**Keywords:** IoT, climatic variables, spectral signature, remote sensing, pithomycotoxicosis, spectroradiometry, Sentinel-2

## Abstract

Spores from the fungus *Pithomyces chartarum* are commonly found on Azorean pastures. When consumed by cattle along with the grass, these spores cause health issues in the cattle, resulting in animal suffering and financial losses. For approximately two years, we monitored meteorological parameters using weather stations and collected and analyzed grass samples in a laboratory to control for the presence of spores. The data confirmed a connection between meteorology and sporulation, enabling the prediction of sporulation risk. To detect the presence of spores in pastures rather than predict it, we employed field spectrometry and Sentinel-2 reflectance data to measure the spectral signatures of grass while controlling for spores. Our findings indicate that meteorological variables from the past 90 days can be used to predict sporulation, which can enhance the accuracy of a web-based alert system used by farmers to manage the risk. We did not detect significant differences in spectral signatures between grass with and without spores. These studies contribute to a deeper understanding of *P. chartarum* sporulation and provide actionable information for managing cattle, ultimately improving animal welfare and reducing financial losses.

## 1. Introduction

The sporulation of the fungus *Pithomyces chartarum* is increasingly common in Azorean cattle pastures. Sporulation is used by fungi as a means of surviving harsh environments and spreading to new areas [1]. The presence of the spores on the pastures leads to their ingestion by cattle, which has health implications for the animal, resulting in a negative economic impact [2,3]. The effects of *P. chartarum* spores have been known and reported since 1999 in cattle in the Azores islands (Portugal) [2]. Pithomycotoxicosis, commonly called facial eczema, is the intoxication caused by sporidesmin, a mycotoxin produced by spores of the fungus *Pithomyces chartarum*. The severity of the intoxication is proportional to the number of spores [4]. Sporidesmin is a potent hepatotoxin that causes pericholangitis and occlusion of the bile ducts in the liver causing jaundice, photosensitivity, loss of milk production, reduced productivity, and in extreme cases, the death of the animal.

Currently, the Regional Veterinary Laboratory (LRV), located on the island of Terceira (Azores, Portugal), performs analysis and spore counting of pasture samples brought to them at a cost of €2.50 per sample. However, spore counting only confirms the existence of the problem, providing information on its density; it does not provide a warning, and therefore does not allow preventive measures to be taken before sporulation occurs. Furthermore, it takes 10 to 14 days between the ingestion of the toxin and the appearance of the first clinical signs of photosensitivity [2,5]. This late manifestation leads farmers to use supplements against the disease in a preventive way without factual knowledge of the likelihood of sporulation. To protect the animals from pithomycotoxicosis, the farmers administer zinc as a feed supplement to the cattle. When ingested, zinc combines with sporidesmin, creating a stable mercaptide and leading to the elimination of the toxin in the autoxidation cycle. Administration is carried out orally, through (a) addition of zinc sulfate (ZnSO_4_) to water (only in animals that are not producing milk, as the supplement reduces water intake), (b) zinc oxide capsules of slow-release zinc (ZnO) (providing protection for four to five weeks), or (c) zinc oxide added to concentrated feed [5]. Consequently, the current practice of zinc supplementation is based on the farmer’s past experience with symptom occurrence, rather than on the factual presence of spores (or the likelihood of sporulation), often resulting in over- or under-administration.

The dynamics of sporulation of *P. chartarum* (PC) appear to be driven by air temperature and relative humidity [5]. Optimum conditions for PC growth are warm, overcast, and showery weather that spans at least three days, or hot fine weather with long overnight dew periods. These conditions allow PC to spend enough time in its temperature and humidity optima to complete sporulation and colonisation in pastures [4]. *P. chartarum* sporulates most actively at grass minimum temperatures above 14 °C [5], with 24 °C being the most active temperature in vitro [6]. The upper temperature limit for fungal growth is 35 °C [7], and moisture is required for sporulation [6,8], with heavy or continuing rainfall reducing its occurrence [8]. Smith et al. [9] proposed that PC spores become less toxic with high rainfall because they become saturated and sporidesmin is water soluble [10]. Brook [6] recorded high PC sporulation with 5.1 to 12.7 mm total rainfall over 1 to 3 days, and Smith et al. [9] recorded high sporulation with 16.3 to 23.4 mm of rain over 3 to 4 days. Mitchell et al. [5] suggested that PC sporulated when rain fell two or more times per week, provided it was not prolonged or heavy. One study found the optimum rainfall range for sporulation to be 5 to 35 mm per week, or an average of 0.7 to 5 mm per day [7].

The effects of this fungus’ spores in the Azores islands have been known for a long time, and one work [2] carried out between 1999 and 2001 documented the phenomenon on two islands. Based on data from two weather stations in Terceira Island and five stations in São Miguel Island, the study reported outbreaks of pithomycotoxicosis associated with a minimum temperature of 16 °C and a minimum relative humidity of 90% for more than 10 consecutive days. August and September were the months with the highest incidence of cases [2]. Studies from other areas of the globe where this phenomenon occurs, such as New Zealand [3,8,9,11], also support these supposed effects of temperature and humidity. One study from the Netherlands documented the first case of pithomycotoxicosis in sheep in October 2019. In that year, August was characterized by high temperatures and dryness, followed by episodes of rainfall and high humidity levels exceeding 90% in late September and early October [12].

Phenological phenomena, such as sporulation, are increasingly analyzed by relating their observed dynamics to variations in environmental drivers [13]. Correlative modelling approaches, frequently based on regression analysis, are particularly useful for understanding these dynamics. These models do not simulate the underlying physiological processes, but instead rely on empirical relationships between climate variables and phenology. This approach has gained increased use in recent years due to the growing availability of phenological observation data and high temporal and spatial resolution weather data from both in situ and ex situ sources [14,15], and has proven valuable in elucidating the relative importance of different meteorological factors and the time periods over which these factors exert their influence [13].

Another well-known approach that could help to identify the presence of sporulation in grassland is the use of remote sensing techniques, either close remote sensing (field spectroradiometry) or remote sensing from air or space. Since *P. chartarum* is a saprophyte [3,11], the presence of dead vegetable matter in the pastures favors the colonization of *P. chartarum*, which may lead to increased spore densities when sporulation occurs [8]. Spectroradiometry is a technique used to measure the properties of the reflectance bouncing off materials (spectral signatures), and is often used to assess vegetation health, including the nutritional status of plants [16]. However, despite its utility in characterizing vegetation, this technique is time-consuming for large-scale applications. The study of large areas of grassland is often carried out with satellite-based and unmanned aerial vehicle (UAV) remote sensing techniques, which follow the same physics fundamentals based on the reflectance spectra of vegetable matter. The biophysical characteristics of grasslands have been extensively studied using Earth observation data over many years, with optical multispectral and hyperspectral images being the primary forms of remote sensing. Multispectral images are the most commonly used, particularly for evaluating architectural traits in crops like green area index and leaf biochemical content, such as chlorophyll, water, and nitrogen [17,18]. RGB images, on the other hand, are mainly used for classification or segmentation tasks, including plant and organ counting, and identifying diseases or pest infestations [18]. These imaging technologies have advanced to the point where they can be used to detect and map crop diseases and infestations, such as weed patches [19], powdery mildew in wheat [20], oil palm diseases, and citrus diseases [21]. Critical parameters of grasslands can also be monitored with multispectral and hyperspectral data, such as the above ground biomass (AGB) [22,23,24], the net primary productivity (NPP) [25,26], and the leaf area index (LAI) [27,28]. However, there is a notable gap in the literature regarding the detection of diseases and infestations in pastures (with livestock), indicating that remote sensing applications in grassland biomes are less common and less efficient compared to those in other vegetation types [29]. Despite this, remote sensing can also be used to identify grassland management practices, such as distinguishing between mown, grazed, or mixed-use grasslands using various classifiers. Popular classifiers for this purpose include support vector machine, random forest, and decision trees [29]. Additionally, remote sensing (RS) techniques are now employed to analyze extensive and intensive grassland management practices [29,30,31]. Using hyperspectral data at appropriate spatial and temporal frequencies allows for the identification, mapping, and description of plant species properties, including non-native grasses [32,33]. However, RS-based vegetation mapping and monitoring are complicated by the high spatial variability caused by terrain, cover, or very small areas. Greater spatial resolution imaging or spectral unmixing modeling, which can be used instead of greater spatial resolution, can handle this heterogeneity [17]. Additionally, to handle this heterogeneity, it is recommended that the spatial resolution is suited to the size of the pasture, i.e., the size of each pixel must be smaller than the size of the pasture. Satellites like the Sentinel-2 (S2) are suitable for the study of Terceira Island. The Sentinel-2 has on board a multispectral sensor with 13 bands, from the visible and near infra-red to the short wave infra-red (SWIR), with a spectral resolution of up to 10 m and a revisit time of 5 days [33].

In this study, we assessed the spectral signatures of grass in pastures on the island of Terceira in the presence and absence of spores using field spectroradiometry and Sentinel-2 reflectance data, aiming to identify differences in the spectra. Simultaneously, we also aimed to understand the environmental conditions driving the sporulation dynamics for the period from 2021 to 2023. This experiment was developed together with the veterinary team at UNICOL-Cooperativa Agrícola, C.R.L. Across the two experimental years, grass samples were collected and analyzed in the laboratory for spore counts, and temperature and relative humidity data were measured in real time by a network of over 60 weather stations installed across the island, most of which communicate via the Internet of Things (IoT) network (LoraWAN of the Azores) [34]. With this study, we aim at contributing to agricultural modernization, helping farmers to better manage livestock and maximize milk production, reduce production costs, and improve animal welfare, as well as monitor the evolution of this phenomenon and improve related scientific understanding.

## 2. Materials and Methods

### 2.1. Study Area Characterization

**Figure 1:**
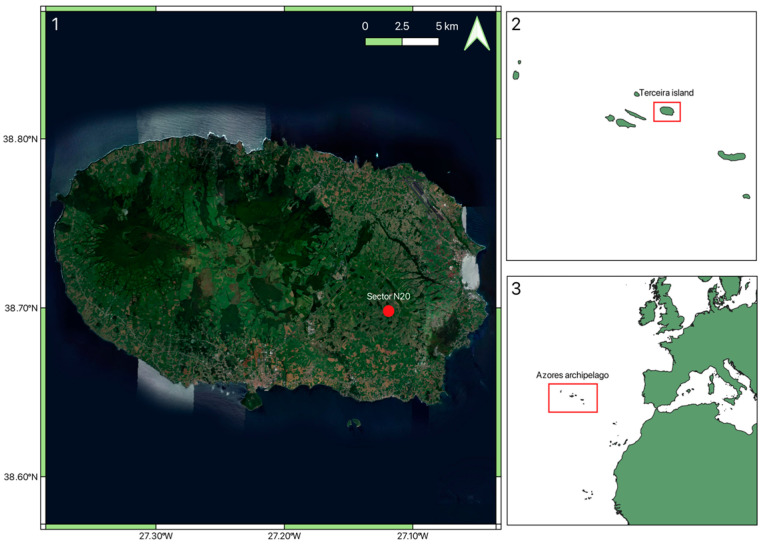
Geographical representation of Terceira Island (**1**), its location within the Azores archipelago (**2**), and within the European continent (**3**). Sector N20, where the study was carried out, is highlighted with a red point.

The Azores archipelago (36 to 39° N, 25 to 31° W), located in the Macaronesia region of the North Atlantic Ocean, is composed of nine islands: Santa Maria, São Miguel, Terceira, Graciosa, São Jorge Pico, Faial, Flores, and Corvo. This archipelago has a semi-permanent subtropical Atlantic anticyclone, frequently called the “Azores High”. It is also referred to as the North Atlantic High-Anticyclone or Bermuda High-Anticyclone, located in a subtropical semi-permanent center of high atmospheric pressure that is responsible for the average meteorological conditions. The climate may be categorized as wet temperate, and because of temperature variations with altitude, in the mountains, the climate can be particularly cold and humid due to heavy rainfall. On each island, the annual average atmospheric moisture content is 80% [2,35]. Terceira Island has a surface area of about 400 km^2^, a perimeter of 90 km, and an elliptical shape, measuring about 29 km in length from east to west and 17.5 km in width from north to south [36]. Its highest point is located in Serra de Santa Bárbara, with an altitude of 1021 m. Terceira’s geographical representation in the Azores archipelago is illustrated in Figure 1.

### 2.2. IoT Network Deployment and Validation

Based on the analysis performed by the AIR Centre team using the data from the existing three meteorological stations installed in Terceira from the Instituto Português do Mar e da Atmosfera (IPMA), a variation of up to 7 °C between the stations was registered. Considering the variance of temperature across the island and the need to have precise and real-time weather data (temperature and relative humidity) to monitor the conditions for sporulation, TERINOV (the Science and Technology Park of Terceira Island) established a network of 59 low-cost IoT (Internet of Things) weather stations and four gateways spread out over Terceira for measuring these parameters continuously in time [37]. IoT sensors are nowadays widely used in precision agriculture since they can monitor critical parameters concerning, for example, crop monitoring, disease prevention, and soil management in real time. Thus, these devices are a tool for increasing productivity in agriculture, precisely enabling the execution of critical tasks at the appropriate time and location [38,39].

The network included 57 R712 Wireless Outdoor Temperature and Humidity Sensors produced by NetVox [40], along with 2 DL-SHT35 Air Temperature and Humidity Sensors for LoRaWAN from DecentLab [41]. Both types communicated via LoRaWAN with the Azores LoRaWAN Network [34]. Some advantages of this protocol included the low-power and long-range communication, allowing connectivity along extensive agricultural areas [42].

The R712 model offers temperature measuring range spanning from −20 °C to 55 °C, with an accuracy of ±1.5 °C at 25 °C. For humidity measurements, it covers a range from 0% to 100% relative humidity (RH), with an accuracy of ±10% RH at 25 °C. These sensors are deployed for temperature and humidity detection outdoors, providing accurate and reliable data for various applications such as outdoor remote monitoring, smart agriculture, urban heat islands, greenhouses, and building automation [43]. The DL-SHT35 sensors provide precise measurements over a wider temperature range (−40 °C to 125 °C) with an accuracy of ±0.1 °C (20 °C to 60 °C) and ±0.2 °C (−40 °C to 90 °C). They also measure humidity from 0% to 100% with an accuracy of ±1.5% RH (0% to 80% RH) and ±2.0% RH (80% to 100% RH).

**Figure 2:**
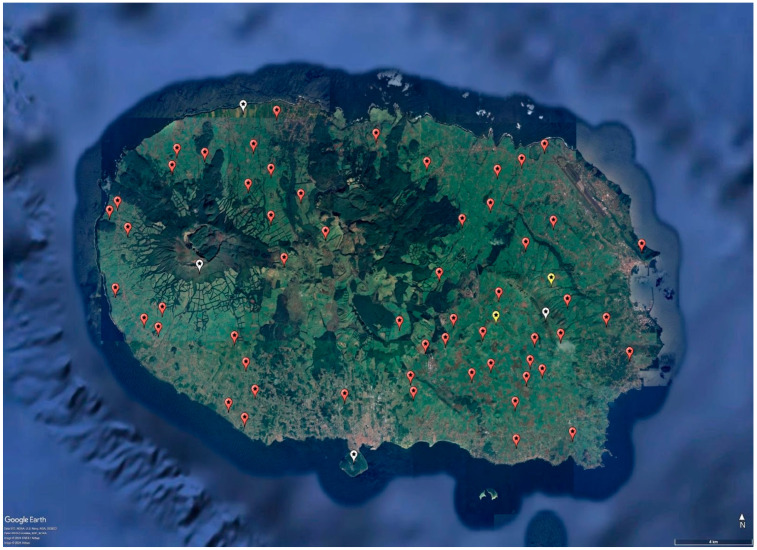
Deployed IoT network configuration. R712 type (red), DL-SHT35 type (yellow), gateways (white). The illustration was developed in the Google Earth Pro app.

The devices were strategically deployed across the pastures on the island. The weather stations were positioned in high-risk areas, where significant sporulation events had been observed over time, and were installed 1–2 m above ground to prevent damage from the presence of livestock in the field, while the gateways were situated at higher elevations in the terrain to provide better coverage of the surrounding area and enhance the performance of the network. Figure 2 illustrates the spatial distribution of the LoRaWAN sensor network across Terceira Island. Weather data from the IoT network began to be collected on 2 July 2022.

**Figure 3:**
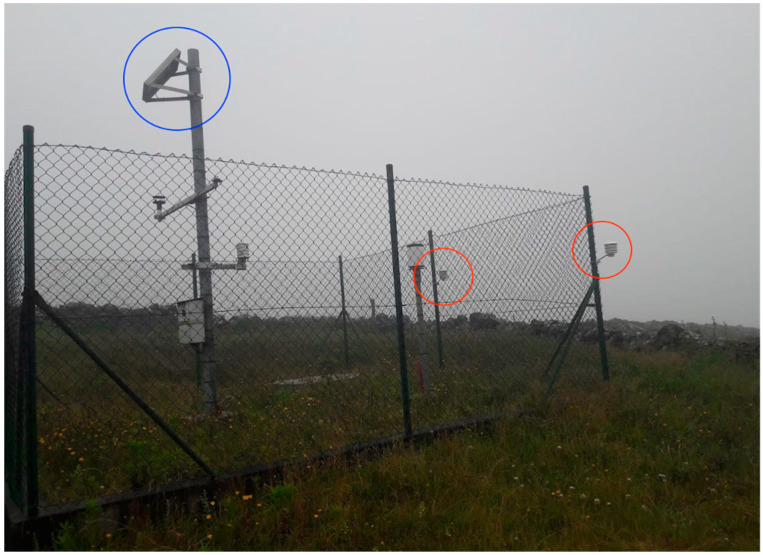
Strategic placement of the IoT sensors used for validation: NVX_R712_00137A1000015794 and NVX_R712_00137A1000015795 (red), and the reference meteorological IPMA station 11217372 (blue).

The data were collected hourly and transmitted to the IoT server via the LoRaWAN protocol, enabling remote access for real-time monitoring of meteorological conditions. Data were stored and accessed using a MySQL database. The Portuguese Institute for Sea and Atmosphere functions as Portugal’s national meteorological institution. To validate the sensor measurements, two low-cost IoT sensors named NVX_R712_00137A1000015794 and NVX_R712_00137A1000015795 were positioned near the IPMA meteorological station, 11217372, which served as a reference station, as illustrated in Figure 3.

**Figure 4:**
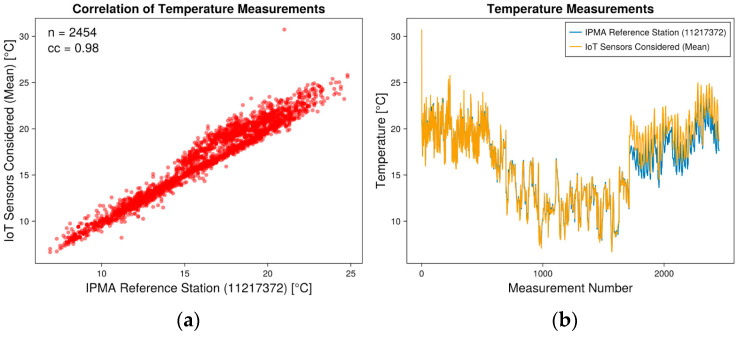
(**a**) Correlation plot between mean temperature measurements from installed IoT stations and the reference IPMA station. (**b**) Temperature values measurements plot.

**Figure 5:**
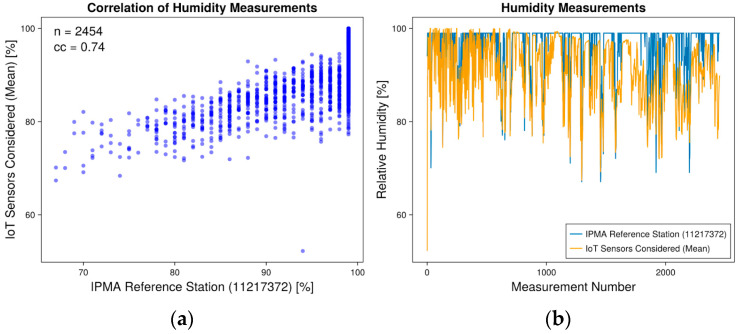
(**a**) Correlation plot between mean humidity measurements from installed IoT stations and the reference IPMA station. (**b**) Humidity values measurements plot.

Data analysis for the validation study was conducted using data collected from 16 July 2022 to 4 July 2023. The meteorological variables considered were temperature and humidity, with primary objectives focused on assessing the reliability, validation, and comparison of newly installed weather stations. To facilitate comparisons, we aggregated data from various stations into a single table based on hourly readings. Subsequently, correlation analyses were performed to assess the similarity between the newly installed stations and the IPMA reference station. Correlation analysis covered 2454 measurements (n). Correlation analyses between mean temperature and humidity values from both stations and the IPMA reference station demonstrated positive correlations. The temperature correlation was strongly positive with a correlation coefficient (cc) of 0.98, while for relative humidity, the coefficient was 0.74, as shown in Figure 4 and Figure 5, respectively. Additionally, the differences (Δ) between pairs of measurements were calculated based on the mean values and the reference station, as illustrated in Table 1. The performance measured proved to be adequate for use within the scope of the project. In both temperature and humidity, the correlation was stronger at the beginning of the measuring process, just after installation. This may be related to the battery levels, which could have led to less accurate measurements over time.

### 2.3. Grass Sampling and Spore Counts

**Figure 6:**
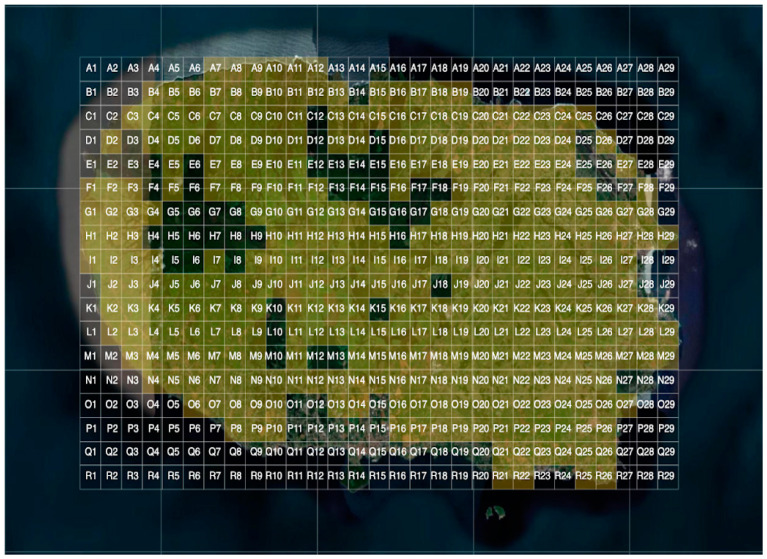
Location of the sectors created on top of Terceira Island’s geographical map to facilitate the identification of the pastures’ location during spore counts. The sectors highlighted in light green are the sectors where meteorological conditions and spore counts were being monitored during the project.

Since the project’s initiation, the nutritionists and veterinarians from Unicol had been gathering grass samples from the locations where livestock graze. For easier identification of the pastures where the sensors were installed and where the samples were taken, we created a grid with squares measuring 1 km × 1 km on top of a geographical map of Terceira’s island. Each square corresponded to a sector and had geographical coordinates. The sectors were ordered alphabetically and numerically (Figure 6). The collected grass samples were identified by location (sector) and subjected to spore counting using a washing method (spores/g of grass) at the Laboratório Regional de Veterinária over the period from June 2021 until December 2023. Grass samples were collected during periods of favorable sporulation conditions, in addition to regular sampling, irrespective of sporulation conditions. Each week, the technicians collected approximately 38 to 40 samples. Spore samples were obtained from 35 different sectors (all inland), with 32 of them directly associated with a meteorological station.

### 2.4. Climatic Variables

**Figure 7:**
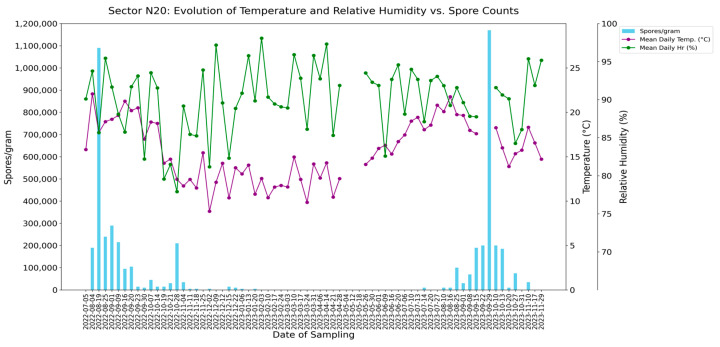
Evolution of mean daily temperature (purple) and mean relative humidity (green) during the period of study (05-07-2022 until 29-11-2023), reflecting two outbreaks of *P. chartarum* spores (05-07-2022 until 29-11-2023).

To investigate the drivers of spatial and temporal variation in the number of spores, we compiled a set of variables that represent climatic conditions at different time periods and have a possible influence on spore formation. Using the local loT sensors, we calculated the mean, maximum, and minimum temperatures (°C) for one day, three days, the week, the month, and the three months prior to each spore sample being taken from the respective sector. The same was performed for the relative humidity (%). A preliminary analysis of the evolution of the temperature and relative humidity in the N20 sector was performed, to check for visual patterns in terms of relation between the rising/falling of the two parameters and the beginning/ending of sporulation periods (Figure 7).

### 2.5. Data Analysis of the Climatic Variables

To analyze the relationships between the number of spore counts and the set of variables, we used a generalized linear mixed model (GLMM). In this way, relevant variables could be identified in order to develop a prediction model. To minimize redundancy among the covariates, we selected those with a variance inflation factor (VIF) below 5 [44]. As the distribution family, we used a negative binomial distribution. We included sectors as a random effect (1|sector) in the model to account for the non-independence of samples from the same sector. For model fitting, we used the R package ‘glmmTMB’ version 1.9.11 [45]. All calculations were performed in R version 4.3.2 [46].

### 2.6. Grass Spectral Signatures Acquisition and Separability Analysis

We organized a field campaign in Terceira Island on the week of 9–14 July 2023 to collect the spectral signatures of the grass in the presence and absence of sporulation in order to conclude whether there were major changes in the spectral signatures between the two scenarios, since sporulation is triggered by the presence of dead vegetation [8]. A preliminary analysis of the results, up until the date regarding the meteorological conditions and the spore counts on the above-mentioned sectors, was used to determine which sectors were going to be analyzed during the field campaign. We chose four sectors that were most affected by sporulation, that is, where the spore counts were above 40,000 spores/g [2], and three sectors that were not affected by the sporulation. These sectors are discriminated between in Table 2 and Table 3.

The spectral signatures were obtained with an ASD FieldSpec 3 spectroradiometer (ASD Inc., Boulder, CO, USA, 1999). This instrument measures in a spectral range from 350 nm to 2500 nm. All spectral signatures were acquired in absolute reflectance mode from approximately 0.5 m height and in the central hours of the day. All of the analyzed sectors had a mix of types of grass. At least 20 spectral signatures were obtained for each grass in each of the sectors. They were then preprocessed to remove the noise observed around certain spectral bands due to the absorption effect of atmospheric water vapor. From the corrected measurements, the average spectral signature of each grass was determined. The spectral signatures acquired were processed using the ViewSpec™ application from the RS3 software [47].

The use of spectroradiometers depends on a technician going to the field and taking measurements locally, making it a time-consuming process limited to small-scale monitoring. We aimed to infer the Sentinel-2 sensor’s suitability for assessing the spectral signatures of the grass remotely, in the presence and absence of spores. A spectral separability analysis of ASD reflectance values for specific wavelength regions, matching the Sentinel-2 (S2) bands, was carried out using the Jeffries–Matusita (J−M) distance, which describes the distance between two statistical distributions or probability density functions. For each wavelength region, the mean reflectance and variance were calculated and fed to Equation (1):

J − M_1,2_ = 2(1 − e^−B1,2^) (1)

In which B_1,2_ represents the Bhattacharyya distance, detailed in Equation (2):

(2) $$B_{1,2}=\frac{1}{8}{\left(\mu_{1}-\mu_{2}\right)}^{T}{\left(\frac{\sum_{1}+\sum_{2}}{2}\right)}^{-1}\left(\mu_{1}-\mu_{2}\right)+\frac{1}{2}\ln\left(\frac{\left|\frac{\sum_{1}+\sum_{2}}{2}\right|}{\sqrt{\left|\sum_{1}+\sum_{2}\right|}}\right)$$

and where the mean vectors of two distributions are represented by $\mu_{1}$ and $\mu_{2}$ and the covariance matrices by $\sum_{1}$ and $\sum_{2}$.

This analysis allowed us to assess the S2 sensor’s capabilities to discriminate between sporulated and non-sporulated grass spectral responses. J–M reaches an asymptote at 2.0, indicating maximum spectral separability between two classes when this value is achieved [48].

### 2.7. Acquisitions of Spectral Signatures of Grass Using Sentinel-2 Reflectance Data

Since Sentinel-2 data offers good temporal coverage with a revisit time of 5 days (Sentinel-2A and 2B) and are freely available, these data are suitable for studying this phenomenon. Due to the the high frequency of clouds in Terceira island, short revisit times can help to obtain a greater volume of cloud-free data in critical periods for the outbreaks of sporulation. Additionally, if the S2’ sensor is appropriated for the discrimination of sporulation in grass, the S2-derived data can be used for creating vegetation indices, thus creating an additional tool for the detection of disturbances in grass infected with spores and the signatures of grass with and without sporulation. We considered four VIs that are commonly used in the literature in this study: the Normalized Difference Vegetation Index (NDVI), the Generalized Difference Vegetation Index (GDVI), the Structure Insensitive Pigment Index (SIPI), and the Enhanced Vegetation Index (EVI).

For this analysis, we focused on the N20 sector, since it was one of the sectors with higher spore counts during 2023, and with more cloud-free data. Analysis in other sectors from the test campaign was not possible due to the high frequency of clouds in the location of these sectors, reflected in the availability of very few cloud-free datapoints during that year. The results of the spore counting and the list of cloud-free Sentinel-2 products during 2023 for the N20 sector are listed in Table 4 and Table 5, respectively. The Sentinel-2 reflectance values were calculated using the mean value for the pixel values within the sector (1 km^2^). The Sentinel-2 passage dates (cloud-free) considered were the ones as close as possible to the grass sampling dates. The interval between the Sentinel-2 passages considered and the grass sampling dates did not surpass 4 days.

## 3. Results

### 3.1. Identification of Climatic Variables for Predicting the Number of Spores

The generalized linear mixed model (GLMM) enabled the identification of variables associated with variation in spore numbers. After removing collinear variables, of the twenty variables for temperature and humidity representing different time periods prior to the sampling events, eight remained to be included in the model (Table 6). Two variables were found to be significantly associated with variation in spore counts (*p* < 0.05), and one had a marginally significant effect (*p* < 0.1). The average temperature 90 days prior to the spore count was positively associated with the number of spores counted, while there was a negative association between the number of spores and the maximum temperature in the previous three days. The average relative humidity in the 90 days prior to the spore count showed a marginal positive effect.

### 3.2. Spectral Signatures of Grass Using ASD Fieldspec 3 Spectrometer

**Figure 8:**
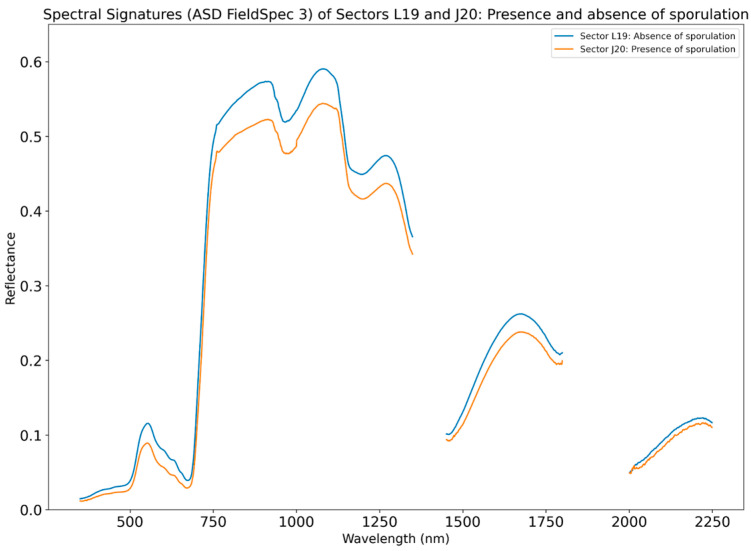
Spectral signatures of the L19 sector (blue) and J20 sector (orange) acquired during the field campaign, in the absence and presence of sporulation, respectively.

During the field campaign, grass samples were collected specifically in the sectors where the spectral measurements were performed, to check for the presence of sporulation. The results of the Regional Veterinary Laboratory showed that on the 14th it was possible to acquire the spectral signature of grass from sector J20 in the presence of spores (40,000 spores/g), and on the same day in the absence of sporulation from sector L19. To analyze differences between the two spectral signatures, we plotted the mean spectral signature of the L19 and J20 sectors (Figure 8). Wavelengths in the range of 1350–1450 nm and 1800–2000 nm were excluded since in these ranges the water vapor present in the atmosphere strongly interacts with the energy reflected from the surface. The results suggest that some slight differences are noticeable in the green, red, and NIR wavelengths, and that the reflectance values from sector J20, in the presence of sporulation, are lower than for sector L19.

Regarding the spectral separability analysis of ASD reflectance values for the wavelength regions that match the Sentinel-2 (S2) bands, we conclude that bands 2 (443 nm), 3 (494 nm), and 4 (560 nm) show moderate separability, suggesting that these bands can be useful for discriminating between sporulated and non-sporulated grass fields. In contrast, band 5 (704 nm) through band 12 (2194 nm) exhibit low separability, indicating that these wavelengths do not provide effective differentiation between the two types of grass fields. However, bands with low separability could still be of use for constructing vegetation indices, potentially enhancing the overall discriminatory power. The results of the J–M analysis are described in Table 7.

### 3.3. Spectral Signatures of Grass Using Sentinel-2 Reflectance Data

**Figure 9:**
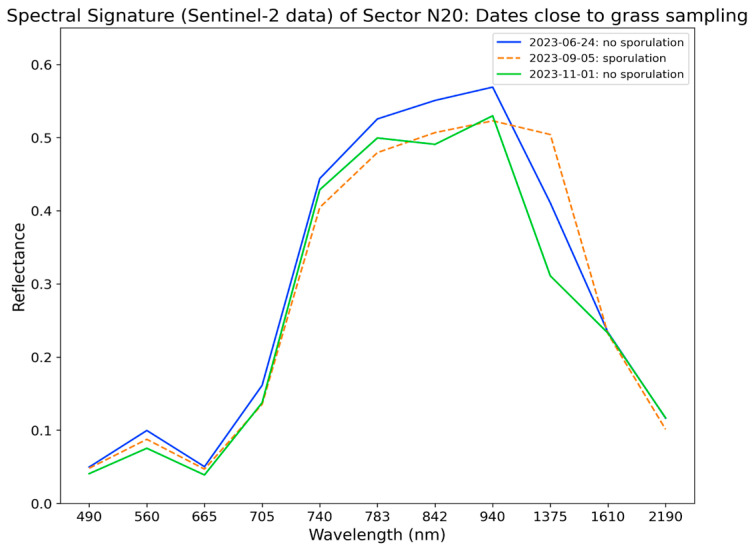
Spectral signatures obtained from Sentinel-2 reflectance data before (blue), during (orange), and after (green) a sporulation outbreak in the N20 sector. Lines that are solid (24-06-2023 and 01-11-2023) represent the dates which were close to a grass sampling that revealed 0 spores/gram, and the dashed line (05-09-2023) represents the date close to a grass sampling that revealed 70,000 spores/g.

In Figure 9, the spectral signatures of the N20 sector, derived from the Sentinel-2 data, are shown. We plotted three spectral signatures: before, during, and after an outbreak of sporulation. We selected the following dates: 24 June 2023, referent to a spore count on 20 June 2023 with 0 spores/g; 5 September 2023, referent to a spore count on 8 September 2023 with 70,000 spores/g; and 1 November 2023, referent to a spore count on 31 October 2023 with 0 spores/g. Ideally, the best day for the analysis of the spectral signature related to a high number of spores was September 29th, with a spore count of 1.170,000 spores/g (see Table 4). However, on this day, there was not a S2 passage by Terceira Island, and it was also not possible to obtain S2 cloud-free data relatively close to this day. Thus, 5 September was considered instead, which was close to a date with a high spore count [2] (see Table 5). Since bands 2, 3, and 4 showed moderate separability, we would expect that the spectral signatures of grass derived from Sentinel-2 reflectance data, with and without spores, would reveal differences in these wavelengths. Analyzing the graph from Figure 9, we conclude that there are slight but not major differences in the reflectance values between the three dates for these wavelengths. In the red-edge region, there are no differences between the three signatures. Between the 1000 nm wavelength and 1610 nm (band 11), even though the signatures are quite similar, the reflectance values from the signature of grass with sporulation are higher than the values from the signatures in the absence of sporulation, especially when comparing with the signature from 1 November 2023.

**Figure 10:**
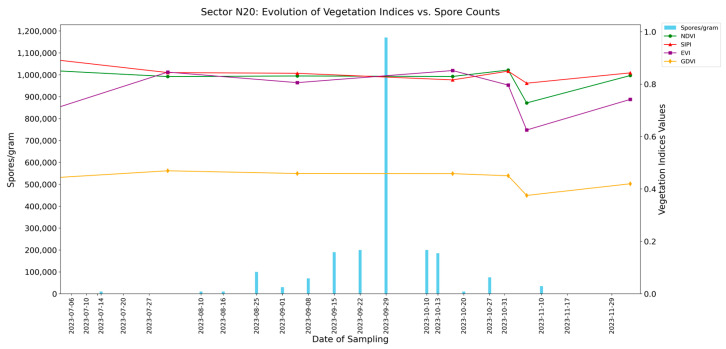
Spore counts in the sector N20 during data collection vs. the vegetation indices NDVI (green), SIPI (red), EVI (purple), and GDVI (orange).

Despite the fact that no distinguished features were observed in the spectral signatures of N20 in the different scenarios, we aimed to analyze the variation in vegetation indices before, during, and after an outbreak of sporulation (Figure 10). Analyzing this graph, we can verify that all index values remained constant regardless of sporulation levels. NDVI, SIPI, and EVI had values around 0.8, while GDVI showed the lowest values at 0.4. At the beginning of November 2023, a generalized drop in all indices was observed.

## 4. Discussion

Comparing the results of the generalized linear mixed model (GLMM) for the identification of climatic variables that are significantly associated with variation in spore counts with the previous studies performed in New Zealand [3,5,8,11] and Terceira [2], we show that environmental parameters over a longer period of time have a relevant influence on sporulation. The impact of temperature and precipitation in the ten preceding days does not capture the whole variation of weather that may impact the growth of *P. chartarum* and the formation of spores, as previously assumed [2]. More specifically, temperature seems to be the most relevant climate variable for sporulation, and relative humidity seems to have a marginal effect. This could be due to the fact that the relative humidity on Terceira Island is consistently high [35], which can also be seen exemplified in Figure 7. The analysis revealed that the higher the maximum temperature in the previous three days prior to spore collection, the lower the number of spores; however, on the other hand, the higher the average temperature 90 days prior to the spore count, the higher the number of spores in the field. The temperature conditions from 90 days prior to sporulation are significant because they can also reflect the typical patterns of the seasons.

In this analysis, we have only included data from the IoT sensors, as these were available for each sector. To improve/refine the model, future work could include other climate variables that are known to influence fungal growth/spore formation. For example, since sporidesmin is soluble in water [10], precipitation may also play a role in sporulation. Some studies indicate that small amounts of rain, increasing the soil moisture, together with warm weather, augment the probability of the occurrence of sporulation. However, it is possible that abundant rain may lead to a loss of toxicity of the spores in colder weather [3,5]. Another climate variable that could be considered is ultraviolet (UV) radiation, which has been proven in a laboratory context to increase sporulation and sporidesmin production in some strains of *P. chartarum* [49].

Regarding the results of the spectral signatures obtained from the ASD Fieldspec 3 spectrometer both in the presence and absence of sporulation, we concluded that no major changes in the signatures were found, suggesting that (1) *P. chartarum* in Terceira can grow both in healthy grass or in grass with dead matter, as verified several times by the technicians during the grass sampling; (2) the amount of dead vegetation in the grass is not significant enough to induce alterations in the spectra of the grass; (3) since farmers in Terceira practice rotational grazing, one hypothesis could be that the cattle itself carries spores through feces [50] or on their hooves, contaminating healthy grass; and (4) the number of measurements with the spectrometer was not enough to ensure that the spectral signatures obtained were representative of the grass in the sectors. Initially, the week of 9–14 July 2023 was chosen since facial eczema cases in Terceira Island typically start appearing in the summertime, and the odds of the weather conditions being favorable were greater for acquiring spectral signatures both with the spectrometer and Sentinel-2 planned passages during those days. We wanted to cover 4 days of tests (days 9, 12, 13, and 14) to cover possible setbacks regarding the weather, most specifically clouds and rain. However, the weather conditions were very unstable during those days, with only the 9 and 14th of July being the most favorable days for spectroradiometer measurements.

Even though the spectral separability analysis for the Sentinel-2 bands in S2 bands after the red edge were not satisfactory, the moderate separability of the other bands led us to consider that Sentinel-2 derived data could be an option to check for unique patterns, especially considering VIs that use those bands as combinations. However, the analysis of the spectral signatures in the presence and absence of spores did not reveal different patterns, and we could verify that the VIs analyzed were incapable of discriminating between sporulation levels. The presence of sporulation did not affect the spectral response, or possibly we were not capable of detecting it, potentially due to the spatial resolution, or due to the lack of data for that time period. Since Terceira Island is very often cloudy, the amount of Sentinel-2 products considered in the analysis led to a reduced dataset. To overcome this fact and to obtain a more robust and complete dataset for the period of study, exploring the use of Sentinel-1 (carrying a radar instrument) data for this study could be a good option, as it also has good temporal coverage and the data are not affected by clouds.

## 5. Conclusions

This study was a step forward in the characterization of *P. chartarum* sporulation on Terceira Island, offering farmers an opportunity for improvement in the management of the milk production of local livestock. The use of IoT weather stations across the island for the monitoring of this phenomenon was the first of its kind to be developed and enabled a more accurate characterization of the climatic variables that play a role in the number of spores. These variables have a relevant influence on spore formation over a longer period and not just over a short period. Regarding the identification of differences in the spectra of grass with and without spores, which was also addressed for the first time, no major differences were found. Biological/ecological studies of the spores’ proliferation, cycle of life, and toxic activity in Terceira’s pastures are needed to support further investigations of this kind, and to open the possibility of including these findings in our prediction model. Future work will consider the use of the variables of precipitation and UV radiation in the generalized linear mixed model, the repetition of spectroradiometer measurements confirming the reflectance values obtained for the grass in the mentioned sectors, as well as performing the same spectral analysis of this study, but with the use of Sentinel-1 derived data.

## Figures and Tables

**Table 1:** Extreme differences between mean values and reference station measurements.

Difference	Temperature (°C)	Relative Humidity (%)
Average	−0.17	−4.46
Min	−9.735	−41.76
Max	2.98	12.09

**Table 2:** Sector names, locations, and geographical coordinates of the pastures with more sporulation considered in the field campaign.

Sector Name	Location	Coordinates of Central Point (Latitude, Longitude)
L8	Escampadouro	38.700307, −27.296928
L19	Granja	38.698152, −27.171611
J20	Paúl (Organic)	38.683481, −27.155684
N20	Paúl	38.698151, −27.118764

**Table 3:** Sector name, location, and geographical coordinates of the pastures with less sporulation considered in the field campaign.

Sector Name	Location	Coordinates of Central Point (Latitude, Longitude)
M28	Porto Martins	38.691933, −27.061997
K28	Cabo da Praia	38.711983, −27.062456
D22	Lajes	38.775122, −27.126245

**Table 4:** Dates of grass sampling in 2023 and respective spore counts for the N20 sector.

Date of Grass Sampling	Spores/g
06-01-2023	5000
13-01-2023	0
20-01-2023	5000
03-02-2023	0
10-02-2023	0
17-02-2023	0
24-02-2023	0
03-03-2023	0
10-03-2023	0
17-03-2023	0
24-03-2023	0
31-03-2023	0
06-04-2023	0
14-04-2023	0
14-04-2023	0
21-04-2023	0
28-04-2023	0
04-05-2023	0
12-05-2023	0
18-05-2023	0
26-05-2023	0
30-05-2023	0
01-06-2023	0
09-06-2023	0
16-06-2023	0
20-06-2023	0
06-07-2023	0
10-07-2023	0
13-07-2023	0
13-07-2023	0
13-07-2023	0
14-07-2023	10,000
20-07-2023	0
27-07-2023	0
10-08-2023	10,000
16-08-2023	10,000
25-08-2023	100,000
01-09-2023	30,000
08-09-2023	70,000
15-09-2023	190,000
22-09-2023	200,000
29-09-2023	1,170,000
10-10-2023	200,000
13-10-2023	185,000
20-10-2023	10,000
27-10-2023	75,000
31-10-2023	0
10-11-2023	35,000
17-11-2023	0
29-11-2023	0

**Table 5:** List of cloud-free Sentinel-2 products in the sector N20, closer to the dates of grass sampling, considered for the study.

Date	Product ID
05-01-2023	S2A_MSIL2A_20230105T124041_N0509_R052_T26SMH_20230105T165558.SAFE
18-01-2023	S2A_MSIL2A_20230118T125031_N0509_R095_T26SMH_20230118T152452.SAFE
12-02-2023	S2B_MSIL2A_20230212T125039_N0509_R095_T26SMH_20230212T163220.SAFE
14-02-2023	S2A_MSIL2A_20230214T124041_N0509_R052_T26SMH_20230214T170217.SAFE
04-03-2023	S2B_MSIL2A_20230304T125039_N0509_R095_T26SMH_20230304T163210.SAFE
20-04-2023	S2B_MSIL2A_20230420T124039_N0509_R052_T26SMH_20230420T132804.SAFE
30-05-2023	S2B_MSIL2A_20230530T124039_N0509_R052_T26SMH_20230530T162231.SAFE
24-06-2023	S2A_MSIL2A_20230624T124041_N0509_R052_T26SMH_20230624T183858.SAFE
27-06-2023	S2A_MSIL2A_20230627T125041_N0509_R095_T26SMH_20230627T185251.SAFE
01-08-2023	S2B_MSIL2A_20230801T125039_N0509_R095_T26SMH_20230801T145651.SAFE
13-08-2023	S2A_MSIL2A_20230813T124041_N0509_R052_T26SMH_20230813T170051.SAFE
16-08-2023	S2A_MSIL2A_20230816T125041_N0509_R095_T26SMH_20230816T171653.SAFE
05-09-2023	S2A_MSIL2A_20230905T125041_N0509_R095_T26SMH_20230905T171456.SAFE
17-10-2023	S2B_MSIL2A_20231017T124039_N0509_R052_T26SMH_20231017T162246.SAFE
01-11-2023	S2A_MSIL2A_20231101T124041_N0509_R052_T26SMH_20231101T165858.SAFE
06-11-2023	S2B_MSIL2A_20231106T124039_N0509_R052_T26SMH_20231106T144526.SAFE
04-12-2023	S2A_MSIL2A_20231204T125031_N0509_R095_T26SMH_20231204T153448.SAFE

**Table 6:** Results of the generalized linear mixed model explaining the spore counts collected across Terceira Island. (.) *p* < 0.1; ** *p* < 0.01; *** *p* < 0.001.

Covariates	Estimate (SE)	z Value	*p*-Value
Intercept	−7.6010 (2.894)	−2.626	0.009 **
Mean humidity 7 days before	−0.0189 (0.039)	−0.482	0.630
Mean temp. 90 days before	0.5936 (0.043)	13.833	<2 × 10^−16^ ***
Mean rel. humidity 90 days before	0.0823 (0.047)	1.755	0.079 (.)
Min. rel. humidity 1 day before	−0.0023 (0.019)	−0.120	0.904
Max. rel. humidity 1 day before	0.0134 (0.029)	0.454	0.650
Max. temp. 3 days before	−0.0432 (0.013)	−3.298	0.001 ***
Min. temp. 3 days before	0.0124 (0.020)	0.632	0.528
Min. rel. humidity 3 days before	0.0192 (0.021)	0.905	0.365

**Table 7:** Spectral separability results of the bands from ASD Fieldspec 3.

Band (Wavelength)	Spectral Separability
Band 2 (443 nm)	1.24
Band 3 (494 nm)	1.27
Band 4 (560 nm)	1.15
Band 5 (704 nm)	1.05
Band 6 (740 nm)	0.20
Band 7 (781 nm)	0.13
Band 8 (834 nm)	0.14
Band 8A (864 nm)	0.16
Band 11 (1612 nm)	0.21
Band 12 (2194 nm)	0.12

## Data Availability

The data presented in this study are available on request from the corresponding author.
